# Platycodin D potentiates proliferation inhibition and apoptosis induction upon AKT inhibition via feedback blockade in non-small cell lung cancer cells

**DOI:** 10.1038/srep37997

**Published:** 2016-11-29

**Authors:** Ting Li, Xin Chen, Xiuping Chen, Dik Lung Ma, Chung Hang Leung, Jin Jian Lu

**Affiliations:** 1 State Key Laboratory of Quality Research in Chinese Medicine, Institute of Chinese Medical Sciences, University of Macau, Macao, China; 2 Department of Chemistry, Hong Kong Baptist University, Kowloon Tong, Hong Kong, China

## Abstract

AKT is the frequently overexpressed and constitutively active kinase within NSCLC cells and recognized as a promising target for NSCLC treatment. However, AKT inhibition relieves the feedback inhibition of upstream receptor tyrosine kinases (RTKs) that may weaken the efficiency of AKT inhibitors. Platycodin D (PD), isolated from widely-used traditional Chinese medicine *Platycodonis Radix*, is now found to remarkably enhance the anti-proliferative effect of AKT inhibitors. In this study, combinatorial activity of AKT inhibitor MK2206 and PD on cell proliferation, apoptosis and related signaling were disclosed. Long-term AKT inhibition induced up-regulation of RTKs, including EGFR and HER-2. Co-treatment of MK2206 with PD could abolish this feedback survival through decrease of EGFR, HER-2, and p-AKT, and profound inhibition of 4E-BP1, leading to an amplified anti-proliferative and apoptotic activity in NSCLC cells. Similarly, feedback activation in response to reduction of AKT expression by small interfering RNA (siRNA) was also blocked by PD and apoptotic effect was further enhanced. Thus, PD potentiated proliferative inhibition and apoptotic induction of both AKT inhibitor and siRNA. These findings also reveal the limitations of suppressing feedback-regulated pathways by monotherapy and establish a mechanistic rationale for a novel combination approach targeting AKT for the treatment of NSCLC.

Lung cancer is the most common incident cancer and the chief cause of cancer death worldwide^1^,^2^,^3^. Non-small cell lung cancer (NSCLC) is one of two main types of lung cancer, representing approximately 80–85%. A large number of clinical data demonstrate that NSCLC has little or no impressive symptoms until it is well-advanced, and it remains poor prognosis^4^,^5^,^6^. Notably, whole-genome sequencing and large-scale omics techniques provided a better understanding of tumorigenesis and tumor progression at the molecular level empowering the development of molecularly targeted drugs for precision treatment of NSCLC^7^. For example, the epidermal growth factor receptor tyrosine kinase inhibitors (EGFR-TKIs) (*e.g*., gefitinib, erlotinib, and afatinib) have been successfully utilized and serve as the first-line therapy for late-stage NSCLC patients harboring EGFR-positive mutation^8^,^9^,^10^. However, these therapies are not always curative. Evidence from clinical treatment show that some patients fail to response to EGFR-TKI within 1 year after the treatment is initialized and ultimately develop progressive disease whichmainly due to the occurrence of a second-site EGFR mutation, *i.e*. EGFR T790M^11^,^12^,^13^. In addition, among the patients with wild type EGFR which account for more than 50% of NSCLC patients, docetaxel- or cisplatin-based chemotherapy remains the first-choice treatment strategies regardless of significant adverse effects^14^. Thus, it is imperative to develop a novel therapeutic strategy for both EGFR mutant and EGFR wild type NSCLC.

The serine/threonine-specific protein kinase AKT, also called protein kinase B, functions as a central signaling node within cells of higher eukaryotes in response to stimuli-activated receptor tyrosine kinases (RTKs) and cytokines to control survival, proliferation, and metabolism^15^. Cancer genetic studies have uncovered AKT as a retroviral oncogene at the core of cancer progression and is frequently overexpressed and constitutively active in NSCLC^16^,^17^,^18^. Therefore, targeting AKT is recognized as an opportunity to fight against tumor complexity and genomic heterogeneity via this central, common oncogenic driver which is essential for the treatment of NSCLC^19^,^20^. Over the past decade, several inhibitors targeting AKT have been developed and displayed promising anti-cancer activity in NSCLC, such as ATP-competitive protein kinase inhibitors, inhibitors of phosphatidylinositol-3,4,5-trisphosphate binding, and other allosteric inhibitors^21^,^22^,^23^. Specifically, MK2206, an orally allosteric small molecule pan-inhibitor of AKT targeting AKT1/2/3, becomes the first AKT inhibitor to enter clinical evaluation in 2008 (NCT00670488). Although MK2206 possesses promising anti-cancer effect in preclinical studies and is well tolerated in phase I clinical trial, no available data from clinical trials till now suggests that it could achieve prolonged survival of selected patient population^24^. Lately, AKT inhibition has been reported to trigger a feedback activation of survival signals, which would contribute to the disappointing clinical results^24^. In this paper and elsewhere, we and others have shown that while the AKT inhibitor effectively suppressed AKT activity, it also increases the protein abundance of several RTKs, such as EGFR, HER-2 and HER-3, *etc*.^25^,^26^,^27^,^28^. Consequently, the release of feedback upon AKT suppression would compromise the anti-cancer efficacy of AKT inhibitors, making a combination treatment that designd to block this negative feedback an attractive strategy to enhance the efficacy of AKT-targeted therapy.

Platycodin D (PD), a triterpenoid saponin isolated from a widely-used traditional Chinese medicine *Platycodonis Radix*, exerts potent anti-cancer activity against many cancer cell lines, including NSCLC cells^29^,^30^,^31^. In our preliminary study, we have observed that PD could decrease the level of EGFR, an all-important upstream RTK of AKT pathway and an attractive target for NSCLC therapy, indicating a potential inhibiton of negative feedback by AKT inhibitors. This finding encourages us to combine an AKT inhibitor with PD. In this present study, we developed a novel and potential combination approach that targeting AKT by co-treatment of MK2206 with PD for the treatment of NSCLC cells. This combination revealed a fully abolishment of the feedback survival stimulated by the AKT inhibitor through the decrease of EGFR, HER-2, and p-AKT, as well as a profound inhibition of 4E-BP1, resulting in amplified anti-proliferative and apoptotic effect in NSCLC cells.

## Results

### The AKT inhibitor MK2206 induced a feedback regulation of EGFR and HER-2

**Figure 1:**
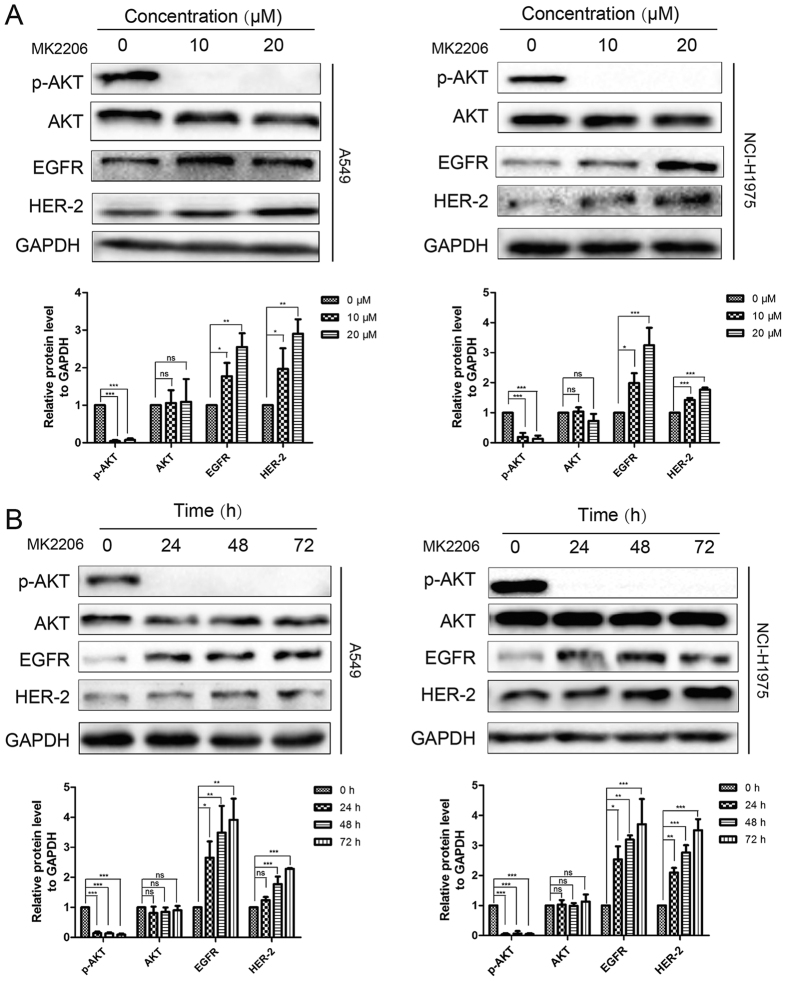
The AKT inhibitor MK2206 induced feedback regulation of EGFR and HER-2. A549 and NCI-H1975 cells were treated with indicated concentrations of MK2206 for 48 h or 10 μM for different time points. Cell extracts were analyzed for the levels of EGFR, HER-2, p-AKT, and AKT by western blot analysis.

MK2206 is a highly selective allosteric AKT inhibitor via binding to the pleckstrin-homology domain of AKT. We firstly treated human EGFR wild-type A549 and EGFR T790M mutant NCI-H1975 NSCLC cells with 10 or 20 μM of MK2206 in different time periods. MK2206 persistently inhibited the phosphorylation of AKT after 48 h treatment at both 10 and 20 μM or after 10 μM treatment at 24, 48, and 72 h (Fig. 1A,B). EGFR and HER-2, the upstream cell surface RTKs of AKT, could transduce growth and survival signals through the forming of dimerization. Recently, it is found that suppression of AKT results in up-regulation of EGFR, HER-2, *etc*.^27^,^28^. As shown in Fig. 1A, both the expression levels of EGFR and HER-2 increased by MK2206 after 48 h treatment in both A549 and NCI-H1975 cell lines. Consistently, the different time (24, 48 and 72 h) exposure of MK2206 at 10 μM to cells yielded a similar up-regualtion of EGFR and HER-2 protein levels (Fig. 1B). These results indicated that inhibition of AKT led to the relief of feedback regulation of EGFR and HER-2.

### PD enhanced the anti-cancer effect of MK2206 in NSCLC cells

**Figure 2:**
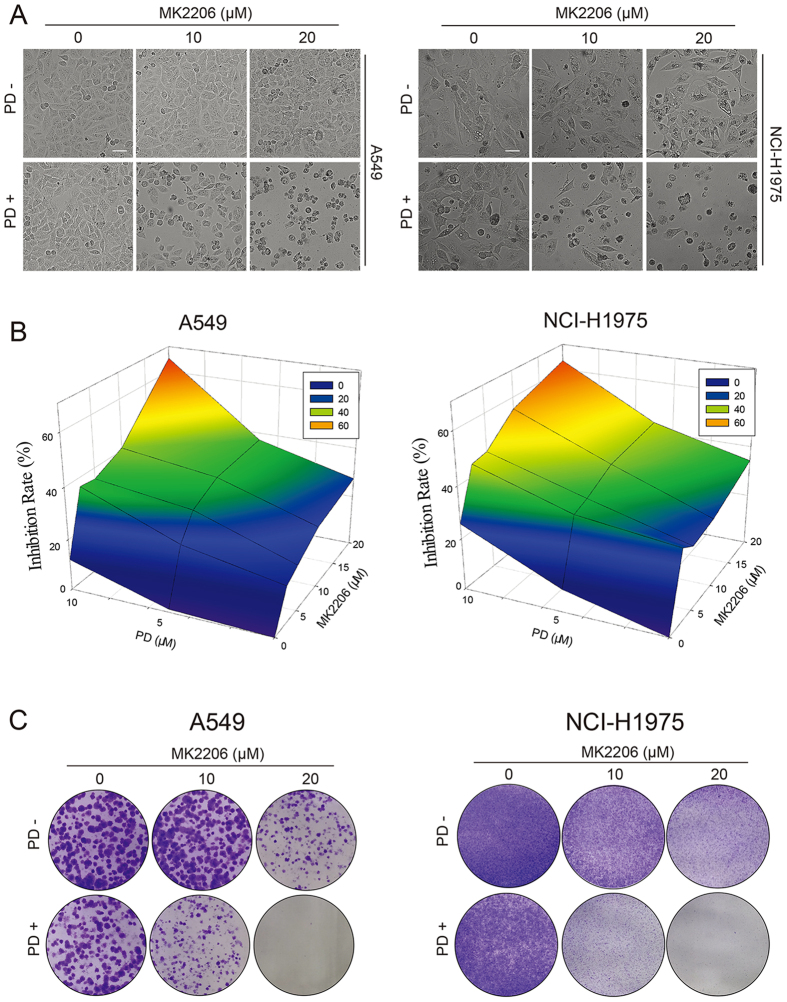
PD enhanced the anti-cancer effect of MK2206 in NSCLC cells. (**A**) A549 and NCI-H1975 cells were treated with indicated concentrations of MK2206 for 48 h with or without PD (10 μM). The cell morphologic changes were observed by In Cell Analyzer 2000 and typical images were presented. Scale bar: 50 μm. (**B**) Cells were treated with indicated concentrations of MK2206 and PD for 48 h, and the cell viability was evaluated by MTT assay and analyzed by SigmaPlot. (**C**) Cell colony formation activity were detected.

By screening a great deal of natural products, PD was found to be a candidate that could be used in combination with the AKT inhibitor. As revealed by the cell morphologic observation, MTT assay detection and colony formation evaluation in Fig. 2, co-treatment with MK2206 and PD obviously heightened the anti-proliferation activity and suppressed colony formation of A549 and NCI-H1975 cells compared to that of each single compound treatment. Treated with MK2206 (10 μM or 20 μM) and PD (10 μM) together, the cells began to crease, then turned into the round shape and shed off. However, no observable morphology changes of the cells could be found after treatment with either single compound (Fig. 2A). Besides, the cell proliferation of A549 and NCI-H1975 were also obviously inhibited through the co-incubation of MK2206 and PD after 48 h, with inhibition rates increasing from 27.5% (20 μM MK2206) or 12.1% (10 μM PD) to 65.2% (20 μM MK2206 + 10 μM PD) in A549 cells, and 34.3% (20 μM MK2206) or 25.8% (10 μM PD) to 63.5% (20 μM MK2206 + 10 μM PD) in NCI-H1975 cells, respectively (Fig. 2B). In addition, the colony formation activity of cells exposed to both MK2206 and PD was further suppressed when compared to that of the cells exposed to MK2206 or PD alone (Fig. 2C). Based on the aforementioned results, this combination strategy may be effective in treatments of different genotypes of NSCLC.

### PD potentiated apoptosis induction by MK2206 in NSCLC cells

**Figure 3:**
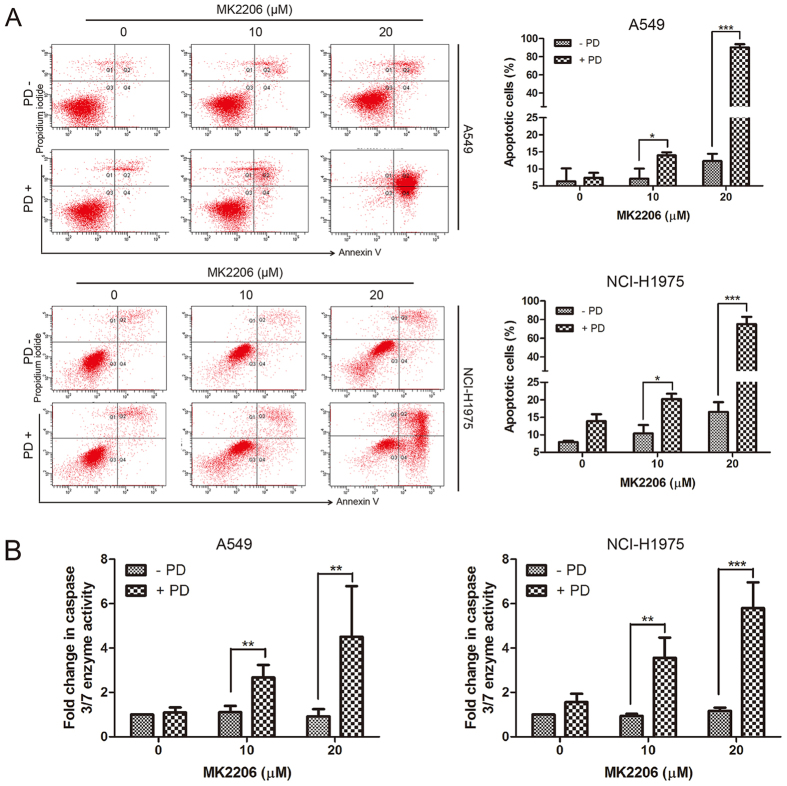
PD potentiated apoptosis induced by MK2206 in NSCLC cells. (**A**) After treated with indicated concentrations of MK2206 for 48 h with or without PD (10 μM), apoptotic cells were stained with Annexin V/PI and analyzed by flow cytometry. Results were shown as mean ± SD for three independent assays. *P < 0.05, **P < 0.01, and ***P < 0.001. (**B**) After treated with indicated concentrations of MK2206 for 48 h with or without PD (10 μM), the enzyme activities of caspase 3/7 were measured.

To detect whether the intensive proliferative inhibition was due to the enhanced induction of cell apoptosis by the combination treatment, A549 and NCI-H1975 cells were incubated with MK2206, PD or both compounds for 48 h and the percentages of apoptotic cells were measured by Annexin V/PI staining assay. As shown in Fig. 3A, co-treatment of these compounds obviously increased apoptotic cell percentages from 7.1% (10 μM MK2206) to 14.0% (10 μM MK2206 + 10 μM PD) or from 12.3% (20 μM MK2206) to 90.4% (20 μM MK2206 + 10 μM PD) in A549 cells, while the cell apoptosis percentages increased from 10.4% (10 μM MK2206) to 20.2% (10 μM MK2206 + 10 μM PD) or from 16.5% (20 μM MK2206) to 75% (20 μM MK2206 + 10 μM PD) in NCI-H1975 cells. Neither MK2206 nor PD treated alone in A549 and NCI-H1975 cells induced obvious apoptosis. To further dissect apoptosis, the activation of caspase 3/7 enzymes activity, biomarkers of apoptosis^32^, was found to be remarkably enhanced after combined treatment of MK2206 and PD in these two cell lines (Fig. 3B). Taken together, apoptosis was involved in the enhanced anti-cancer effect of MK2206 and PD in NSCLC cells.

### MK2206 and PD abrogated the survival signals induced by the inhibition of AKT

**Figure 4:**
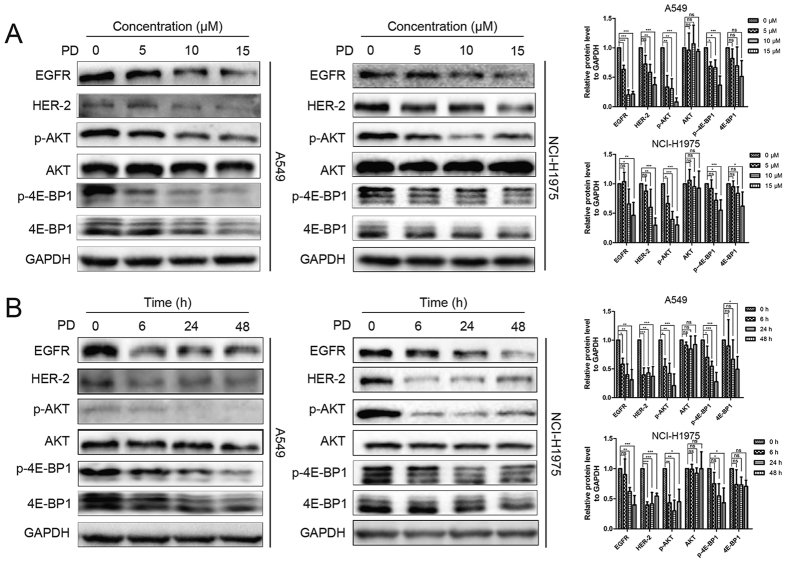
PD decreased EGFR and HER-2, accompanied by the inhibition of AKT and 4E-BP1. Cells were treated with indicated concentrations of PD for 48 h. Cell extracts were analyzed for the levels of EGFR, HER-2, p-AKT, AKT, p-4E-BP1 and 4E-BP1 by western blot analysis (**A**). Cells were treated with 10 μM PD for different time points. Cell extracts were analyzed for the levels of EGFR, HER-2, p-AKT, AKT, p-4E-BP1 and 4E-BP1 by western blot analysis (**B**).

**Figure 5:**
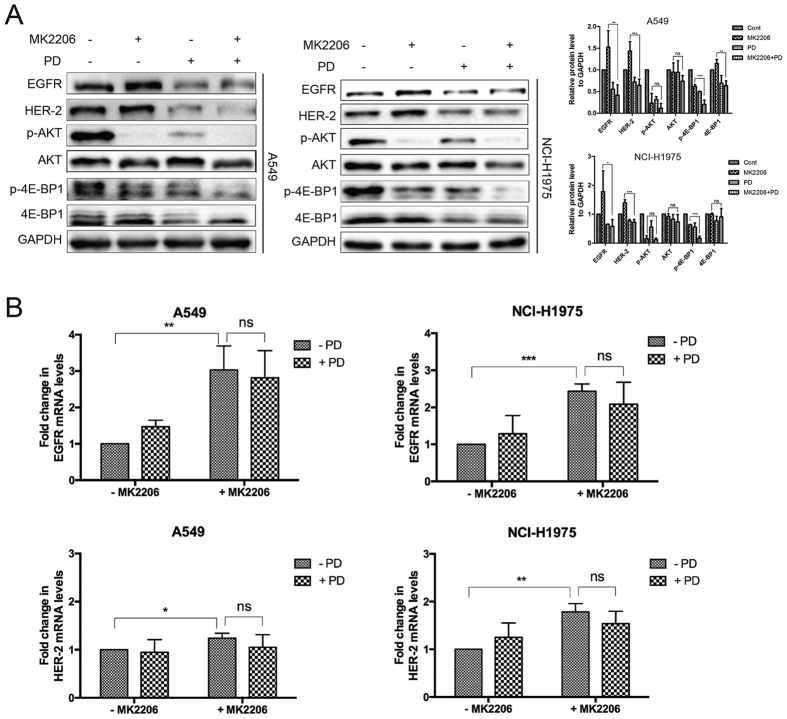
MK2206 and PD abrogated the survival signals induced by inhibition of AKT. Cells were treated with indicated concentrations of MK2206 for 48 h with or without PD (10 μM). Cell extracts were analyzed for the levels of EGFR, HER-2, p-AKT, AKT, p-4E-BP1 and 4E-BP1 by western blot analysis (**A**). Cells were treated with indicated concentrations of MK2206 for 48 h with or without PD (10 μM). The mRNA levels of EGFR and HER-2 were studied with RT-PCR assay (**B**).

In the preliminary study, we uncovered that PD could down-regulate EGFR. As showed in Fig. 4A,B, PD treatment decreased the protein level of EGFR and HER-2, followed by suppression of AKT and 4E-BP1 phosphorylation in A549 and NCI-H1975 cells. To further evaluate the activity of MK2206 is potentiated by PD, the activity on regulation of EGFR and HER-2 and the consequential downstream signals were then investigated by co-treatment of MK2206 and PD. Accordingly, after the addition of PD, MK2206-induced up-regulation of EGFR and HER-2 was reversed (Fig. 5A). 4E-BP1 has been recognized as a key integrator of survival signals and was thus a potential therapeutic target^33^. Crippling the phosphorylation of 4E-BP1 exerted biologic importance in the growth of tumor cells. As shown in Fig. 4, treatment with PD markedly caused a reduction of 4E-BP1 phosphorylation. Moreover, combined treatment of MK2206 and PD caused a further decrease of 4E-BP1 phosphorylation (Fig. 5A), therefore abrogated the consequential survival signals, and enhanced the apoptotic response. These results suggested that PD decreased EGFR and HER-2 levels after treatment with MK2206 together, followed by the decrease of p-AKT, against NSCLC cells irrespective their EGFR status. Besides, amplified apoptosis by combination of MK2206 and PD, at least in part, due to suppression of 4E-BP1 phosphorylation, providing a combination strategy to inhibit AKT/4E-BP1 pathways as well as RTKs.

Although PD reduced EGFR and HER-2 protein expression even in presence of AKT inhibition, it was unclear whether PD exerted transcriptional control of EGFR and HER-2. Therefore, we measured the transcription levels of EGFR and HER-2 by using RT-PCR assay. As shown in Fig. 5B, our results favored that AKT inhibition by MK2206 increased EGFR and HER-2 levels both in mRNA and protein levels. It appeared that PD did not affect the up-regulation of EGFR and HER-2 in the transcription level (Fig. 5B). These results together suggested that PD-controlled EGFR and HER-2 expression is predominantly at the protein level.

### SiRNA of AKT and PD suppressed the proliferation, induced apoptosis and decreased the phosphorylation of 4E-BP1

**Figure 6:**
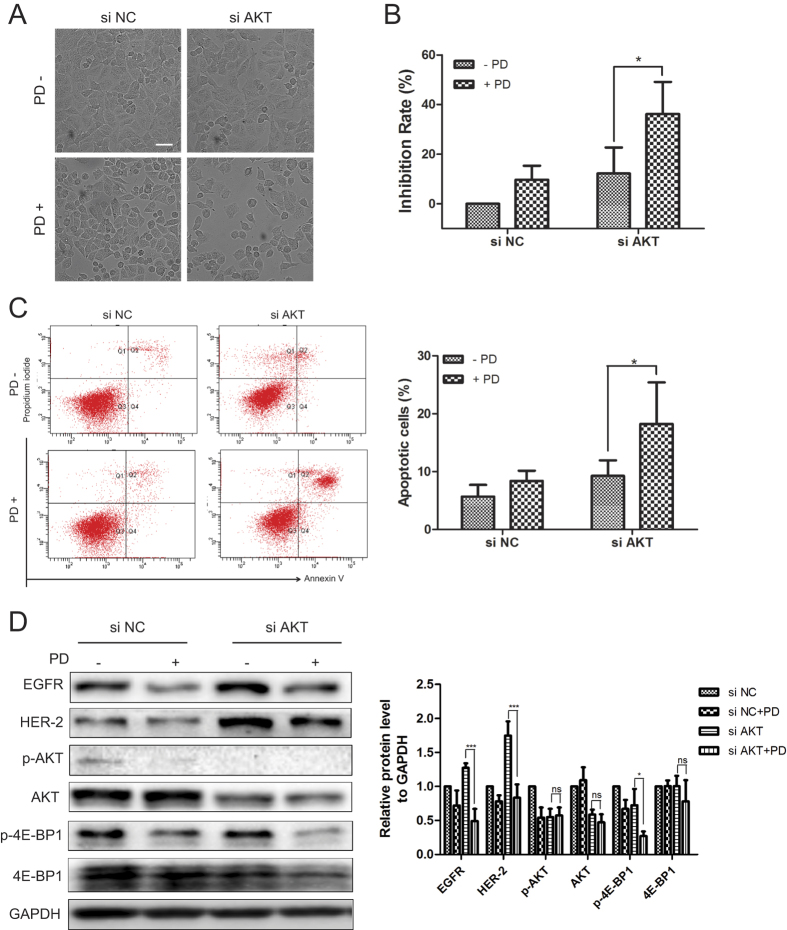
SiRNA of AKT and PD suppressed the proliferation, induced apoptosis and decreased phosphorylation of 4E-BP1. After knockdown of AKT, cells were treated with 10 μM PD for 48 h. (**A**) The cell morphologic changes were observed by In Cell Analyzer 2000 and typical images were presented. Scale bar: 50 μm. (**B**) The cell viability was evaluated by MTT assay. (**C**) Apoptotic cells were stained with Annexin V/PI and analyzed by flow cytometry. Results shown were mean ± SD for three independent assays. (**D**) Cell extracts were analyzed for the levels of EGFR, HER-2, p-AKT, AKT, p-4E-BP1 and 4E-BP1 by western blot analysis.

These data revealed that inhibition of AKT by MK2206 and PD may be promising to obviously affect NSCLC. To explore the feasibility and expand the application of this therapeutic strategy, we knocked down AKT by a specific siRNA in A549 cells. Consistent with results of combination of MK2206 and PD, after knockdown of AKT, PD treatment induced amplified anti-proliferative and enhanced the apoptotic effect (Fig. 6A–C). The inhibition rates were changed from 12.1% (without PD) to 36.2% (with PD), and the apoptotic cells increased from 9.2% (without PD) to 18.2% (with PD) within AKT knockdown cells. As showed in Fig. 6D, AKT knockdown also caused a feedback loop, including up-regulation of EGFR, and HER-2 as well. PD was able to decrease the levels of EGFR and HER-2 upon AKT inhibition, and sufficient to inhibit phosphorylation of 4E-BP1. Thus, PD relieved the feedback activation upon inhibition of AKT either by AKT allosteric inhibitor or by AKT siRNA, which may due to the inhibition of 4E-BP1 function.

## Discussion

Incidence and mortality of NSCLC has been increasing, making it the leading cause of cancer death and a challenge all over the world^2^. Even though 56 to 74% patients with advanced EGFR-mutant NSCLC respond to EGFR-TKI with a 10- to 14-month median progression-free survival, acquired resistance is still occurred soon after treatment initiation and seems endless^34^,^35^. In addition, most of NSCLC patients harbor wild type EGFR and could not benefit from the treatment of EGFR-TKI^36^. Recent evidence suggests that AKT kinase is constitutively active and has implication with cell death escape and drug resistance in NSCLC^19^, making AKT an attractive target and potential utility for the treatment of different genotypes of NSCLC. However, a feedback loop induced by AKT-targeted cancer therapy has been disclosed and a corresponding combination strategy remains to be found.

AKT inhibition is reported to induce up-regulation of RTKs in HER-2-amplified breast cancer cells^27^. We now showed that this feedback responses referring to induction of EGFR, and HER-2, was also elicited upon the pathway (AKT inhibitors) or oncogene (siRNA) blockade in both EGFR T790M mutant and EGFR wild type NSCLC cell lines (Figs 1 and 6). Allegedly, the increase RTKs levels correlates with decrease cell death in cancer treatment. Moreover, the expression of EGFR and HER-2, instead of the other members of RTK superfamily, have important functions in the development of NSCLC^37^. With overexpressing EGFR and HER-2, tumor cells show aggressive cell growth due to the increased potential for EGFR/HER-2 hetero-dimerization and signaling^38^. These findings elucidate a new step that restrain of EGFR and HER-2 may enhance the anti-proliferative effect of AKT inhibitors in NSCLC. On the basic of these observations, we combined the AKT kinase inhibitor MK2206 with PD, which was able to decrease the protein level of EGFR and HER-2, in order to block this feedback activation and enhanced the therapeutic efficiency.

PD, a major saponin isolated from the classical traditional Chinese medicine *Platycodonis Radix*, displays potent anti-cancer activity both *in vitro* and *in vivo* as a single agent or combination constituent against multiple cancer including NSCLC^39^,^40^,^41^. In the present study, PD enhanced the anti-cancer activity with MK2206 in NSCLC cells irrespective of their genotypes (Fig. 2). Additionally, no obviously enhanced toxic effect was induced by these two compounds in the non-cancer L-O2 cells (data not shown). We also found that this enhanced anti-cancer effect was attributed to the profound induction of apoptosis (Fig. 3). PD has been studied to possess inhibition potential on EGFR in human breast cancer cells^42^. Present study directly compared the samples of EGFR and HER-2 mRNA and protein expression and observed that the amount of EGFR and HER-2 mRNA mediated by AKT inhibition did not alter in the presence of PD, indicated that the possible mechanism underlying PD-induced EGFR and HER-2 depletion may not be associated with the transcriptional regulation. Pretreatment of MG132 (a proteasome inhibitor) could partially, but still far from completely, recovered PD-reduced EGFR protein expression (data not shown). It is possible that the protein synthesis, rather than increased proteasome activity, played a key role in PD-mediated down-regulation of EGFR and HER-2. Previous studies have also identified that the mature EGFR and HER-2 are likely dependent on Hsp90, a member of the heat shock protein/chaperone family assisting in the folding of synthesized proteins^43^,^44^. It seemed that PD-down-regulated the EGFR and HER-2 protein expression was similar to the Hsp90 inhibitor in NSCLC cell lines^43^. Although this is the first observation of PD as a possible HSP90 inhibitor to regulate the expression of EGFR and HER-2, further evidence is still needed.

Due to the complexity of signaling network, better understanding of mechanism involved would be useful for constituting combined regimen. PI3K/AKT and ERK signaling share many downstream targets, comprising regulators of cell cycle, cell proliferation, and cell growth^33^. The survey results presented here support the recent studies showing that the inhibition of AKT signaling also cause the compensatory activation of ERK pathway (data not shown)^45^. This is somewhat surprising, given that combined treatment of MK2206 and PD induced an enhanced p-ERK levels, but was still more effective in repressing cell proliferation and triggering apoptosis than the single agent alone. This suggests that integrators of AKT and ERK pathway may be affected by the combination therapy. In particular, 4E-BP1 is identified as pivotal downstream target of both AKT and ERK pathways and plays an important part in mediating the effects of these pathway in cancer^33^,^46^. 4E-BP1, as a translation repressor, binds to the translation initiation factor eIF4E, and then suppresses the activation of cap-dependent translation, which could also be reversed by hyperphosphorylation of 4E-BP1^47^. Moreover, phosphorylated 4E-BP1 is found to be closely related to tumor growth in different phenotypes of cancers, including NSCLC^33^,^48^. This is supported by our finding that decrease of p-4E-BP1 by the combination strategy was sufficient to suppress the growth of both NSCLC cells, regardless of the activation of ERK (Figs 5A and 6D).

**Figure 7:**
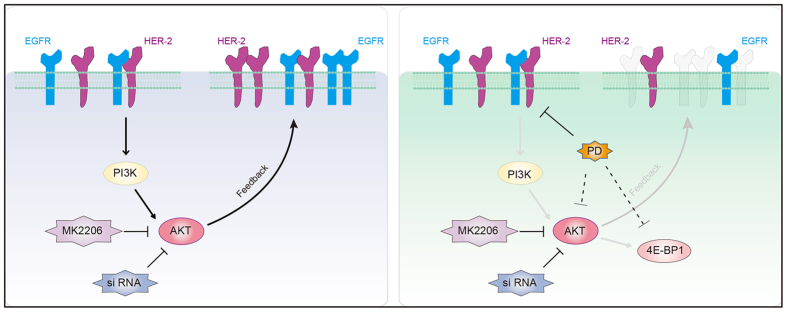
A schematic representation of signaling responses to AKT inhibition and PD combination. AKT inhibition by pharmacological and siRNA mediation activates a feedback increase in EGFR and HER-2. PD could decrease EGFR and HER-2, and inhibit the phosphorylation of AKT and 4E-BP1. Upregulated EGFR and HER-2 upon AKT inhibition were reversed by PD, accompanied by the blockade of AKT signaling and downstream 4E-BP1, causing the enhanced response to AKT inhibition. PD, platycodin D; siRNA, Small interfering RNA.

Taken together, we have found that the combined an AKT inhibitor and PD overcomes the feedback activation of the AKT pathway and leads to blockage of AKT/4E-BP1 function in cell proliferation and apoptosis of NSCLC with different genotypes. Our findings showed that effective treatment required an approach that targets multiple nodes in the pathway to maintain downregulation of the RTK/PI3K/AKT signal (Fig. 7), providing the rationale for novel and potential combination strategy targeting AKT to improve therapy through blocking feedback activation of signaling pathways in NSCLC.

## Material and Methods

### Reagents

MK2206 (PubChem CID: 24964624) and PD (PubChem CID: 162859) were purchased from Selleck Chemicals (Houston, TX, USA) and Best-Reagent (Chengdu, Sichuan, China), respectively. Compounds were dissolved in dimethylsulfoxide (DMSO) as the stock solutions of 40 mM and stored at −20 °C. Then compounds were further diluted in culture media with a final DMSO concentration not exceeding 0.1% before each experiment. 3-[4,5-Dimethyl-2-thiazolyl]-2,5-diphenyltetrazolium bromide (MTT), DMSO, phenylmethanesulfonyl fluoride were obtained from Sigma (Saint Louis, MO, USA). The protease and phosphatase inhibitor cocktail was bought from Thermo Fisher Scientific Inc. (Waltham, MA, USA). Radio immunoprecipitation assay buffer (RIPA buffer) and crystal violet were purchased from Beyotime Biotechnology (Shanghai, China). The primary antibodies against AKT, p-AKT (Ser473), EGFR, HER-2, 4E-BP1, p-4E-BP1 (Ser65), and GAPDH as well as the anti-rabbit IgG HRP-conjugated secondary antibody were all purchased from Cell Signaling Technology (Beverly, MA, USA). Lipofectamine® 2000 Reagent was purchased from Invitrogen (Carlsbad, CA, USA). siRNAs were purchased from Shanghai GenePharma (Shanghai, China) and primers were prepared by Invitrogen Life Technologies (Shanghai, China).

### Cell culture

Human lung cancer A549 and NCI-H1975 cells were all obtained from ATCC (Rockville, MD, USA) and cultured in RPMI 1640 medium (Gibco, Grand Island, NY, USA) containing 10% fetal bovine serum (FBS, Gibco, Carlsbad, CA, USA), 100 U/mL penicillin, and 100 μg/mL streptomycin (Gibco, Grand Island, NY, USA). The cells were maintained at 37 °C in a humidified incubator with an atmosphere containing 5% CO_2_. Exponentially growing cells were used in the experiments.

### MTT assay

Cells were plated in 96-well plates at a density of 6,000 cells per well for 24 h. Then cells were treated with different concentrations of compounds diluted in a medium containing 0.5% FBS for another 48 h. The supernatant was then removed and loaded with 100 μL per well of MTT solution (1 mg/mL). The cells were maintained in a humidified environment for 4 h. Cell proliferation was determined by addition of 100 μL DMSO and 10 min shake in the dark to solubilize formazan. The absorbance at 570 nm was recorded using a SpectraMax M5 microplate reader (Molecular Devices, Sunnyvale, CA, USA).

### Observation of morphologic changes

Cells were transferred to 96-well plates at a density of 6,000 cells per well. After treatment with compounds diluted in a medium containing 0.5% FBS for 48 h, the cellular morphology was observed with an Axiovert 200 inverted microscope (Zeiss, Oberkochen, Germany).

### Colony formation assay

Cells were transferred to 6-well plates at a density of 500 cells per well. After treatment with compounds diluted in a medium containing 0.5% FBS for 48 h, the supernatant was then removed and cultured in a medium containing 10% FBS for one week with renewal every two days. And then cells were stained by crystal violet and observed with an Axiovert 200 inverted microscope (Zeiss, Oberkochen, Germany).

### Caspase 3/7 activity assay

Caspase 3/7 activity assay kits (Cell Signaling Technology, Inc., Beverly, MA, USA) were utilized to study the caspase activities in accordance with the manufacturer’s instructions. Cells were plated into 96-well plates and cultured for 24 h. Cells were incubated with indicated concentrations of MK2206 with or without PD (10 μM). Cells were then lysed on ice for 5 min and caspase 3/7 assay reagent (200 μL) was added into each well and incubated for 1 h. Luminescence was detected using a microplate reader (Perkin Elmer, 1420 Multilabel Counter Victor3, Wellesley, MA, USA).

### Western blot analysis

Cells were lysed in the lysis buffer, which contains RIPA (50 mM Tris pH 7.4, 150 mM NaCl, 1% NP-40, 0.5% sodium deoxycholate, sodium orthovanadate, and sodium fluoride), PMSF, and the protease inhibitor, Cocktails. After incubation for 20 minutes on ice, the lysis buffer containing cellular contents were centrifuged at 4 °C, 12,500 rpm for another 20 minutes. And then the cell supernatants containing the aimed protein were obtained and quantified using a BCA^TM^ protein assay kit (Pierce, Rockford, IL, USA). Equal amount of cell lysate was subjected to SDS-PAGE, transferred onto polyvinylidene fluoride membranes, and then blocked with 5% nonfat milk in TBST (20 mM Tris, 500 mM NaCl, and 0.1% Tween-20) at room temperature for 2 h with continuous rocking. The membranes were probed with specific primary antibodies overnight at 4 °C. The membranes were then washed with TBST three times for 15 min each and incubated with anti-rabbit IgG with HRP-linked secondary antibody in TBST at room temperature for 1 h. The specific protein bands were visualized using an ECL advanced Western blot detection kit (GE Healthcare, Uppsala, Sweden).

### Annexin V/PI staining

The Annexin V-FITC/PI apoptosis detection kit was used to detect the apoptotic cells according to the manufacturer’s instruction. Cells were seeded into a 12-well black culture plate at a density of 20,000 cells per well, and maintained in the incubator for 24 h. Cells were then treated with different concentrations of compounds diluted in the medium that contains 0.5% FBS. After 24 h incubation, cells were harvested, washed twice with cold PBS and re-suspended in 100 μL binding buffer. After 15 min incubation in the dark at room temperature, the collected cells were added with 5 μL Annexin V-FITC and 10 μL PI and then analyzed by a flow cytometer (BD FACS Canto^TM^, BD Biosciences, SF, USA).

### Gene silencing experiments

Cells were seeded in a 6-well plate the day before transfection at 50% confluency. Lipofectamine® 2000 Reagent was used to transfect cells with the specific target sequences of AKT siRNA (sense 5′-UGCCCUUCUACAACCAGGATT-3′, antisense 3′-TTACGGGAAGAUGUUGGUCCU-5′). Cells were transfected with the AKT siRNA (10 nM) for 30 h according to the manufacture’s protocol, and then treated with PD (10 μM) for an additional 48 h. The relative protein levels were measured by western blot analysis.

### RT-PCR assay

The mRNA expressions of indicated genes were studied with RT-PCR assay. Total RNA was extracted from cells using TRIzol reagent (Invitrogen, USA). The extracted total RNA was reverse transcribed into single stranded cDNA using a SuperScript^TM^ III first strand cDNA synthesis kit (Toyobo, Japan). Real-time PCR was performed using SYBR Green PCR Master Mix (Life Technologies, USA) on a Stratagene Mx3005P multiplex quantitative PCR system (Agilent Technologies, USA). GAPDH was used as an internal control, and 2^−ΔΔCT^ values were normalized to control levels. Each sample was analyzed in triplicate. The primers used in this study are presented. The primers of HER-2: 5′-GAAGATCTTTGGGAGCCTGG-3′ (forward), 5′-ACTTGGAGCTGCTCTGGCT-3′ (reverse); EGFR: 5′-GAGAGGAGAACTGCCAGAA-3′ (forward), 5′-GTAGCATTTATGGAGAGTG-3′ (reverse); GAPDH: 5′-GCGACACCCACTCCTCCACCTTT-3′ (forward), 5′-TGCTGTAGCCAAATTCGTTGTCATA-3′ (reverse) were prepared by Invitrogen Life Technologies.

### Statistical analysis

All experiments were repeated at least three times. Data were expressed as mean ± SD. Statistical analysis was performed using analysis of variance (ANOVA) of Graph Pad Prism 5 (GraphPad Software, Inc., CA, USA) and 3D Mesh of SigmaPlot 12.5 (Systat Software Inc. SF, USA). *P value < 0.05, **P value < 0.01, ***P value < 0.001, and the “ns” stands for “not statistically significant”.

## Additional Information

**How to cite this article**: Li, T. *et al*. Platycodin D potentiates proliferation inhibition and apoptosis induction upon AKT inhibition via feedback blockade in non-small cell lung cancer cells. *Sci. Rep*. **6**, 37997; doi: 10.1038/srep37997 (2016).

**Publisher's note:** Springer Nature remains neutral with regard to jurisdictional claims in published maps and institutional affiliations.
